# Physical-Biological Coupling in the Western South China Sea: The Response of Phytoplankton Community to a Mesoscale Cyclonic Eddy

**DOI:** 10.1371/journal.pone.0153735

**Published:** 2016-04-18

**Authors:** Lei Wang, Bangqin Huang, Kuo-Ping Chiang, Xin Liu, Bingzhang Chen, Yuyuan Xie, Yanping Xu, Jianyu Hu, Minhan Dai

**Affiliations:** 1 Key Laboratory of the Coastal and Wetland Ecosystems, the Ministry of Education, Xiamen University, Xiamen, China; 2 State Key Laboratory of Marine Environmental Science, Xiamen University, Xiamen, China; 3 Institute of Marine Environmental Chemistry and Ecology, National Taiwan Ocean University, Keelung, Taiwan; Fisheries and Oceans Canada, CANADA

## Abstract

It is widely recognized that the mesoscale eddies play an important part in the biogeochemical cycle in ocean ecosystem, especially in the oligotrophic tropical zones. So here a heterogeneous cyclonic eddy in its flourishing stage was detected using remote sensing and *in situ* biogeochemical observation in the western South China Sea (SCS) in early September, 2007. The high-performance liquid chromatography method was used to identify the photosynthetic pigments. And the CHEMical TAXonomy (CHEMTAX) was applied to calculate the contribution of nine phytoplankton groups to the total chlorophyll *a* (TChl *a*) biomass. The deep chlorophyll *a* maximum layer (DCML) was raised to form a dome structure in the eddy center while there was no distinct enhancement for TChl *a* biomass. The integrated TChl *a* concentration in the upper 100 m water column was also constant from the eddy center to the surrounding water outside the eddy. However the TChl *a* biomass in the surface layer (at 5 m) in the eddy center was promoted 2.6-fold compared to the biomass outside the eddy (p < 0.001). Thus, the slight enhancement of TChl *a* biomass of euphotic zone integration within the eddy was mainly from the phytoplankton in the upper mixed zone rather than the DCML. The phytoplankton community was primarily contributed by diatoms, prasinophytes, and *Synechococcus* at the DCML within the eddy, while less was contributed by haptophytes_8 and *Prochlorococcus*. The TChl *a* biomass for most of the phytoplankton groups increased at the surface layer in the eddy center under the effect of nutrient pumping. The doming isopycnal within the eddy supplied nutrients gently into the upper mixing layer, and there was remarkable enhancement in phytoplankton biomass at the surface layer with 10.5% TChl *a* biomass of water column in eddy center and 3.7% at reference stations. So the slight increasing in the water column integrated phytoplankton biomass might be attributed to the stimulated phytoplankton biomass at the surface layer.

## Introduction

Most of the tropical and subtropical zones in the open ocean are permanently stratified. Nutrients that are essential for the growth of marine phytoplankton are abundant below the thermocline, but normally they have to rely on diffusion to reach the sun-lit euphotic zone to be utilized by phytoplankton [1,2]. As the rate of diffusion is slow, phytoplankton growth is usually limited by the availability of nutrients such as nitrogen, phosphorus and iron. As a result, tiny unicellular phytoplankton (i.e. pico-phytoplankton) are successful inhabitants in these vast ecosystems due to their superiority (e.g. large surface-to-volume ratio) in coping with trace amounts of essential nutrients [3]. Eddies, one of the common mesoscale events in the tropical and subtropical marine ecosystems, provide a means by which nutrients below the thermocline can be relatively quickly delivered into the upper euphotic zone [2]. Phytoplankton biomass and community structure within the cyclonic eddies often respond noticeably to the enhanced nutrient supply induced by eddies, which is usually found in the subsurface chlorophyll maximum layer instead of in the upper mixed layer [4,5]. The responses of Chl *a* biomass to the mesoscale cyclonic eddy were concluded as four mainly processes, including eddy advection, doming DCML to the near surface layer, promoting phytoplankton biomass supported by the nutrient pumping and simply convergence of the biomass under the currents [6,7]. The combined action of different processes made it be necessary to study the biological response to the mesoscale eddy in the view of 3D structure.

Not only the response of Chl *a* biomass, the phytoplankton community structure also has an obvious response to the cyclonic eddies. The prosperities of diatoms in cyclonic eddies were widely reported [4,5,8], especially for some larger diatoms species like centric genera, *Rhizosolenia* and *Chaetoceros* [4]. Some studies, however, found that non-diatom eukaryotes (mainly refer to haptophytes) were the major players responding to eddies [9–11]. Even the pico-phytoplankton acted differently in the cyclonic eddies. There was an increasing in the relative amount of *Prochlorococcus* spp. and a decreasing in *Synechococcus* spp. came with rarely diatoms or dinoflagellates species in the cyclone C1 at BATS [12]. In general, if the cyclonic eddy appeared in an oligotrophic pelagic ecosystem, it was expected that a diatom dominated community would be formed under a large nutrient pulse, while a nano-phytoplankton dominated community would be generated from a continuous low nutrient supply [5].

Phytoplankton biomass and production are very low in most surface water of the tropical South China Sea (SCS), where inorganic nutrients are usually scarce [13–16]. The oligotrophic environment in the euphotic zone of the SCS, essentially a “microbial loop” [17] or “microbial food web” [18], is a result of the permanent stratification of the water column [19]. In the western SCS in summer, strong wind stress from the prevailing southwesterly monsoon, accompanied with an eastward jet, results in the area having a high frequency of eddy appearance [20,21]. Usually a pair of eddies are formed together, including an anti-cyclonic eddy in the south and a cyclonic eddy in the north [22,23]. This pair of eddies are founded rhythmically in late June or early July and last to October, being totally dissipated at the end of October. Their mature stage is between August and September [24–26].

In the SCS, studies on mesoscale cyclonic eddies mainly focused on physical oceanographic processes and circulation modeling [24,27,28], but rarely on biological responses. The distribution of phytoplankton species using microscope was described and they were associated with a cold eddy in the western SCS [25]. And the coupling of the growth rate of pico-phytoplankton and the micro-zooplankton grazing rate in the cyclonic eddy was investigated [29] in the same survey as us. But these studies did not give the full size information of the phytoplankton community, and did not cover the entire eddy as a process study. According to the scarcity of the physical-biological process study in the western SCS and the various responses of phytoplankton to the mesoscale eddy in the global scale, we focused on the biological responses of phytoplankton biomass and community composition to a cyclonic eddy in the western SCS. The primary objective of our study was to ascertain how the different phytoplankton groups response to the episodic nutrient pumping in the cyclonic eddy, both in vertical and horizontal scale. And what role did the phytoplankton community play in the physical-biological coupling processes in the western SCS.

## Methods

### Study area and sampling

A cruise was carried out in the western SCS (11–13°N; 109.5–113°E) on board R/V “*Dongfanghong 2*” from 2 to 8 September 2007 under the permission of the Ministry of Foreign Affairs of the People's Republic of China. A 3-week life-span cyclonic eddy was observed from 26 August to 11 September in our study area. A cyclonic eddy (hereafter called C2) was present in the north and an anti-cyclonic eddy (AC2) in the southeast and a jet occurred between these two eddies [27,28].

**Fig 1:**
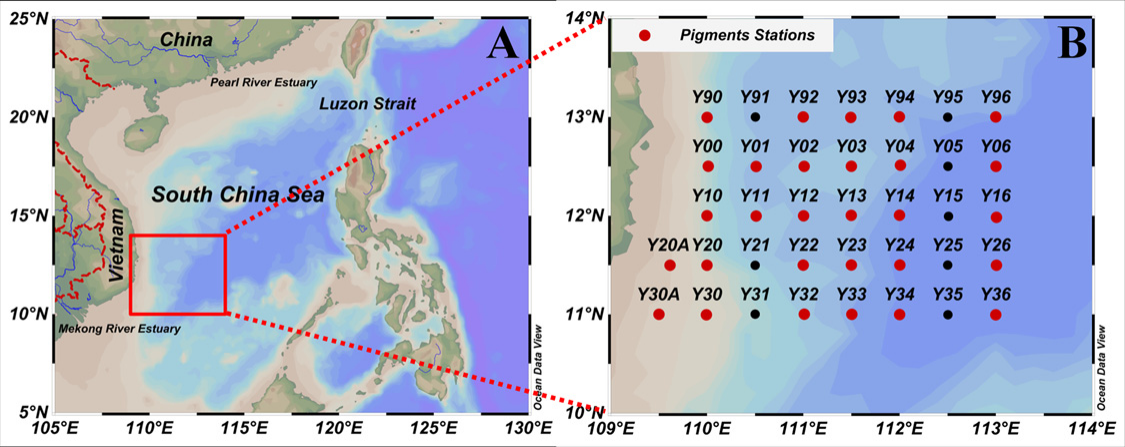
**Maps of the study area (A) and location of sampling sites (B) during the September 2007 cruise.** The red dots indicate the 29 stations where the phytoplankton pigment survey was carried out during the cruise.

High performance liquid chromatography (HPLC) pigments were measured at 29 of the total 37 stations surveyed during this cruise (Fig 1). SeaBird SBE 9/11 and 9/17 Plus CTD systems were deployed to acquire hydrographic measurements. Current velocity was measured using a ship-mounted acoustic Doppler current profiler. To identify the locality of the eddy, the weekly merged data of the sea level anomaly (SLA) were acquired from the French Archiving, Validation, and Interpolation of Satellite Oceanographic (AVISO, http://www.aviso.oceanobs.com/en/data.html) data project. Seawater samples were collected in succession at standard depths (0, 25, 50, 75, 100 and 150 m) and in addition at the deep chlorophyll *a* maximum layer (DCML) using CTD-mounted rosette assemblies with 12 L or 30 L Niskin bottles (General Oceanic Inc.). The DCML was determined by both of the fluorescence and TChl *a* pigment profiles (S1 Fig). The euphotic zone (Z_*eu*_) was calculated using the depth with 1% intensity of surface photosynthetically active radiation (PAR) [30]. The mixed layer depth (MLD) was estimated according to the ± 0.2°C and 0.03 kg m^-3^ threshold criterion [31]. The stratification index (SI) was calculated according to the density difference normalized to the depth difference (100 m in this study) [32].

### Nutrients

Nutrient samples were filtered through 0.45 μm acetate-fiber filters, then either immediately analyzed onboard or refrigerated at 4°C and analyzed within 24 h using a Technicon AA3 Auto-Analyzer (Bran-Luebbe) [33]. In the present study, nitrate concentrations were reported as the sum of nitrate and nitrite, with a detection limit of 0.1 μmol L^-1^. Nitrate concentrations below the detection limit were deemed as 0.1 μmol L^-1^ for statistical analysis. The detection limit for phosphate was 0.08 μmol L^-1^. Phosphate concentrations below the detection limit were deemed as 0.08 μmol l^-1^ for statistical analysis.

### Photosynthetic pigments and biomass of phytoplankton

Seawater samples (4~16 L) for pigment analysis were filtered onto Whatman GF/F filters (pore size: 0.7 μm) of 47 mm diameter under a gentle vacuum (<150 mmHg). The filters were wrapped with aluminum foil and frozen stored in liquid nitrogen on board. When transported to the laboratory the frozen samples were placed into a freezer (-80°C). The pigment concentrations were detected using HPLC following the standard method [34,35]. The frozen filter was soaked in 2 ml N, N-dimethylformamide and extracted in a freezer (-20°C) for 2 h [36]. The extractions were then filtered through Whatman GF/F filters (pore size: 0.7 μm) of 13 mm diameter (Swinnex Filter Holder) to clean the debris and then mixed with ammonium acetate solution (1 mol L^-1^) (600 μL: 600 μL). Each mixture was partially injected into an Agilent series 1100 HPLC system fitted with a 3.5 μm Eclipse XDB C_8_ column (100×4.6 mm; Agilent Technologies). Quantification was confirmed using the standards manufactured by the Danish Hydraulic Institute Water and Environment, Hørsholm, Denmark.

The chemical taxonomy program, CHEMTAX, was applied to acquire the relative contributions of taxa to the total chlorophyll *a* (TChl *a*). Thirteen pigment markers were introduced to quantify each fraction of TChl *a* pool of nine phytoplankton classes, including dinoflagellates, diatoms, haptophytes_8, haptophytes_6, chlorophytes, cryptophytes, *Prochlorococcus*, *Synechococcus* and prasinophytes. The ratios of initial inputting pigment to Chl *a* followed the processes used in previous studies [37–41]. Based on the rule of running CHEMTAX [41], successive runs were necessary to gain convergence between input and output ratios (S1 Text).

### Data analysis

We used Pearson’s correlation efficient to identify the correlative relationships between two variables. Independent *t*-tests or Duncan Post-hoc Multiple Comparisons combined with One-Way ANOVA was used to compare the difference between two groups or multiple, respectively.

## Results

### Physical structure of the eddy and adjacent waters

**Fig 2:**
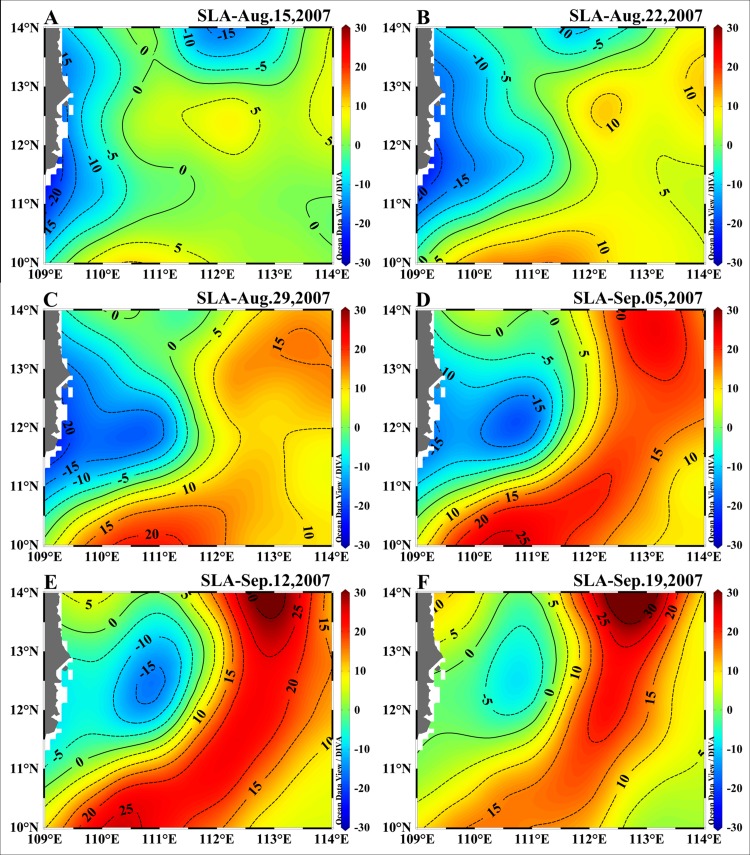
Evolution of the cyclonic eddy C2 in the western SCS during the cruise. The weekly merged data of the SLA product from the French AVISO data project, from 15 August to 19 September 2007.

After a transitory formation stage from 22 to 29 August, the C2 intensified to its mature stage with a steady center (110.8°E, 12.2°N) near Sta.Y12 for about 10 days. During its later stage, the eddy decayed rapidly and was dissipated between 12 to 19 September (Fig 2). Our survey took place when the C2 was in its mature stage.

**Fig 3:**
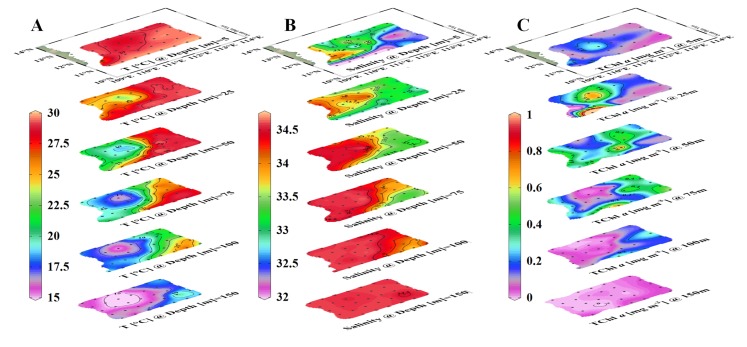
Three-dimensional structures of (A) temperature [°C], (B) salinity and (C) total chlorophyll a [mg m^-3^] in the western SCS during the cruise.

The CTD profile data were utilized to determine the three-dimensional structures of temperature and salinity in the C2 (Fig 3A and 3B). A low temperature and high salinity water (< 20°C, salinity >34.2) rose from 100 m to the near surface zone (25 m) in C2 center. The cold core outcrop (< 25°C) was observed at the 25 m layer with a diameter of about 100~150 km. There was no notable signature of cooler water at the surface layer (5 m), but more distinct water masses could be distinguished based on the salinity distribution. A lower salinity Mekong River Plume Water (salinity <31.5) was observed at the 5 m and 25 m layers in Transect Y3. The plume drifted eastwardly and its tongue extended to 111.5°E. More details concerning the C2’s physical dynamic mechanism had been sufficiently reported [28], as well as the Mekong River plume [13].

### The vertical distribution of TChl *a* biomass

The three-dimensional structure of TChl *a* (mg m^-3^) reflected the cold-core water pumping in C2 center (Fig 3C). The TChl *a* concentration was apparently higher in the center (0.211±0.050 mg m^-3^) than in the adjacent water (0.110±0.047 mg m^-3^) at surface layer, so was at the 25 m layer (0.522±0.153 mg m^-3^ in center, and 0.169±0.085 mg m^-3^ in the adjacent water). However, a reverse pattern occurred from 50 m to 100 m, where the TChl *a* biomass in the center (0.082±0.062 mg m^-3^) was lower than in the surrounding water (0.215±0.168 mg m^-3^). And the TChl *a* was almost vanished at the 150 m layer (<0.020 mg m^-3^) both inside and outside of C2. In the southern boundary of our study area, where the Mekong River plume affected, there was a slightly higher concentration of TChl *a* at the surface layer (0.100~0.200 mg m^-3^). And the plume water penetrated to 20 m layer where a high concentration was observed at Y30 (2.027 mg m^-3^).

**Fig 4:**
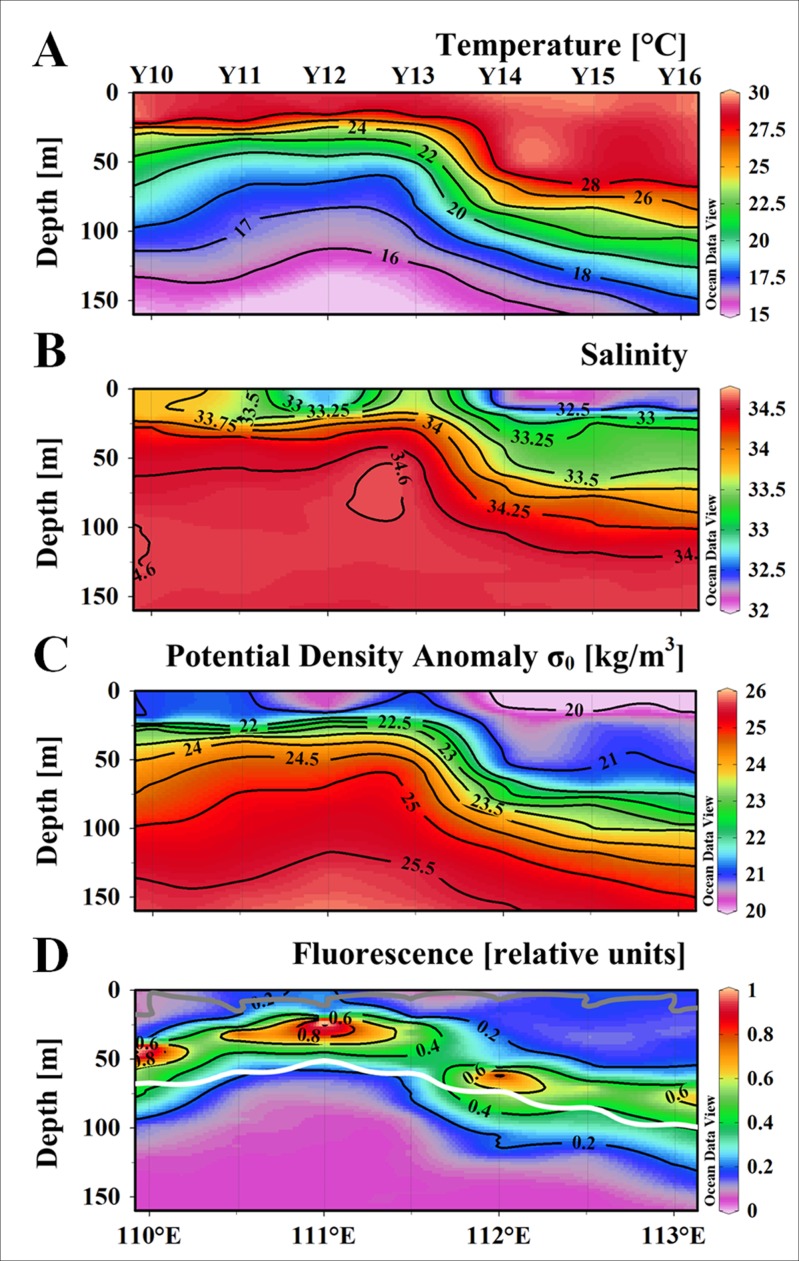
Vertical distribution of hydrologic parameters, nutrients and *in situ* fluorescence along the Y0 during the cruise. (A) Temperature [°C], (B) Salinity, (C) potential density anomaly [kg m^-3^], (D) fluorescence [relative units]. The bright contour denotes the euphotic depth [m], and the gray one denotes the mixed layer depth [m].

**Fig 5:**
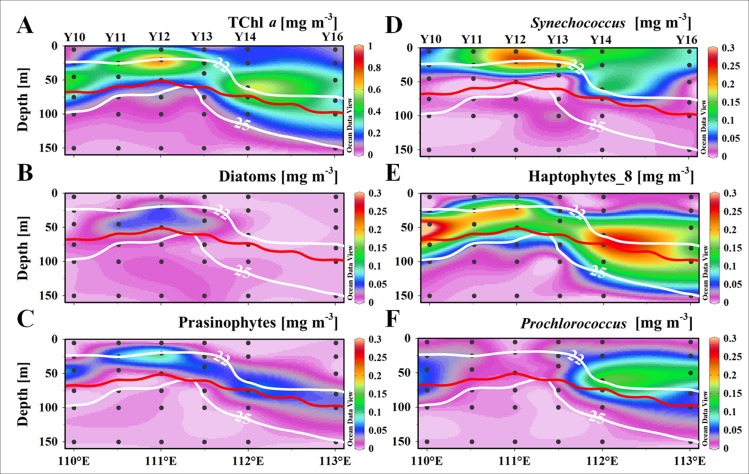
Vertical distribution of TChl *a* and major phytoplankton groups in terms of TChl *a* biomass [mg m^-3^] along section Y0 during the cruise. (A) TChl *a*, (B) Diatoms, (C) Prasinophytes, (D) *Synechococcus*, (E) Haptophytes_8, and (F) *Prochlorococcus*. The bright contours denote the isopycnals bordered between 22.0 kg m^-3^ and 25.0 kg m^-3^, and the intermediated red line is the euphotic depth [m].

In order to better understand the impact of nutrients pumping on phytoplankton community composition in the cyclonic eddy, the vertical distribution of phytoplankton and related environment parameters along Transect Y1 were presented. There was a dome structure at the center of C2 according to the vertical distribution of the temperature, salinity and density along Transect Y1 (Fig 4A, 4B and 4C). The Chl *a* largely followed the doming isopycnal in vertical (Fig 4D). The DCML was almost coincided with the isopycnal of 22.0 kg m^-3^, where was the top of the nutricline. The DCML was at about 50 m at the eddy edges and deeper than 75 m outside the eddy, and was uplifted to about 25 m at the center. In Transect Y1, the TChl *a* maximums at the DCML exceeded 0.740 mg m^-3^ at the center and edge, which was higher than the stations outside the eddy (0.551 mg m^-3^). In contrast, The TChl *a* concentration was low and fairly uniform below the DCML (Figs 4D and 5A).

**Fig 6:**
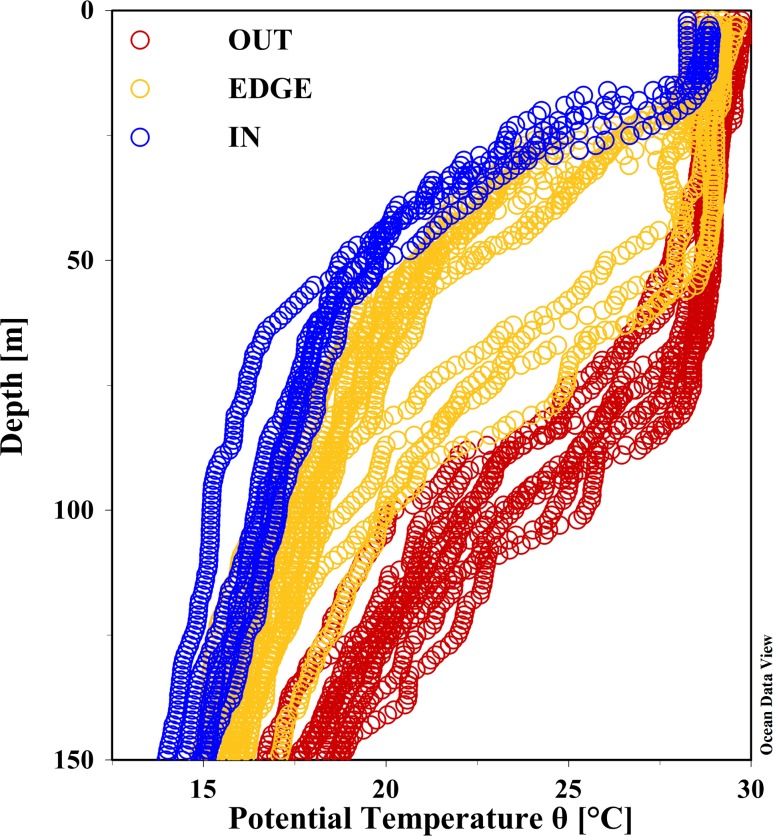
The upper 150 m profiles of potential temperature (°C) for the survey stations to distinguish the three blocks of the C2 (IN, EDGE and OUT).

In order to analyze the difference in the phytoplankton biomass and community structure response to the C2, three blocks were divided according to the profiles of potential temperature (Fig 6), the hydrology parameters (Table 1), the current vectors measured by the underway acoustic Doppler current profiler (ADCP) data [25] (S2 Fig) and the time series SLA variation (S3 Fig). The three blocks were the center of the C2 (IN, five stations), the edge of the C2 (EDGE, twelve stations) and stations outside the eddy (OUT, nine stations). Additionally, vertical distribution, including the water column integrated (upper 100 m), the DCML and the surface layer (at 5m), were introduced to dissect the heterogeneity of phytoplankton composition synthetically among the three horizontal blocks.

**Table 1:** The hydrologic parameters of the three blocks of C2.

Parameters	IN	EDGE	OUT
**SST [°C]**	28.704±0.233^**^	28.984±0.325^**^	29.495±0.347
**MLD [m]**	14.2±1.6	17.2±3.1	20.6±7.5
**SI [kg m**^**-4**^**]**	0.045±0.003^*^	0.042±0.004	0.036±0.008
**Z**_***eu***_ **depth [m]**	63.3±4.9^*^	53.1±9.4^**^	83.4±9.8
***θ* = 22.0 kg m**^**-3**^ **depth [m]**	20.4±3.3^***^	37.3±15.4^***^	79.0±7.5
**Sta. labels**	Y01,Y02,Y11,Y12,Y13	Y90,Y92,Y93,Y94,Y00,Y03,Y04,Y10,Y14,Y20,Y22,Y23	Y96,Y06,Y16,Y24,Y26,Y32,Y33,Y34,Y36
**Number of Sta.**	5	12	9

The three blocks were the center of C2 (IN), the edge of C2 (EDGE) and stations outside C2 (OUT). The parameters included sea surface temperature (SST), mixed layer depth (MLD), the stratification index (SI), the euphotic layer depth (Z_*eu*_) and the depth of the 22.0 kg m^-3^ isopycnal. The OUT group was treated as the control in the multiple comparison using one-way ANOVA.

* implied significant difference in the level of p<0.050

** for p<0.010

*** for p<0.001

For all of the multiple comparisons, the OUT was taken as the control group. The TChl *a* concentration was homogeneous among the three blocks at both the water column integration and the DCML (Table 2). On the contrary, the TChl *a* concentration at the surface layer was 0.211±0.050 mg m^-3^ in IN, which was 2.6-fold higher than in the OUT (p<0.001) and 1.6-fold higher than in the EDGE (p<0.050). The contribution of the TChl *a* concentration at surface to the water column integration was 10.5% in IN, 6.6% in EDGE and 3.7% in OUT, respectively.

**Table 2:** The comparison of TChl *a* biomass and the contribution of major phytoplankton groups to the TChl *a* biomass among the three blocks.

Comparison means	Parameters	IN	EDGE	OUT	IN/OUT	EDGE/OUT
**Water column integration [mg m**^**-2**^**]**	**TChl *a***	20.981±3.710	21.218±5.856	21.644±5.389	1	1
	**Dinoflagellates**	0.327±0.082	0.272±0.132	0.313±0.111	1	0.9
	**Diatoms**	2.402±1.673^***^	0.703±0.519	0.113±0.185	21.3	6.2
	**Haptophytes_8**	7.168±1.805	8.399±3.925	9.591±2.846	0.7	0.9
	**Haptophytes_6**	1.008±0.490	0.928±0.478	1.311±0.329	0.8	0.7
	***Prochlorococcus***	0.988±0.408^***^	2.838±1.920^**^	5.285±0.914	0.2	0.5
	***Synechococcus***	5.422±1.142^*^	3.722±1.803	3.388±0.751	1.6	1.1
	**Prasinophytes**	1.982±0.565	2.240±1.246	1.211±0.684	1.6	1.8
**DCML [mg m**^**-3**^**]**	**TChl *a***	0.522±0.153	0.445±0.143	0.458±0.143	1.1	1
	**Dinoflagellates**	0.009±0.004^*^	0.006±0.002	0.005±0.002	1.8	1.2
	**Diatoms**	0.051±0.074^*^	0.010±0.009	0.002±0.003	25.5	5
	**Haptophytes_8**	0.115±0.066^*^	0.199±0.090	0.242±0.081	0.5	0.8
	**Haptophytes_6**	0.027±0.017	0.019±0.013	0.032±0.015	0.8	0.6
	***Prochlorococcus***	0.018±0.015^**^	0.049±0.045^*^	0.103±0.031	0.2	0.5
	***Synechococcus***	0.184±0.047^***^	0.057±0.054	0.026±0.017	7.1	2.2
	**Prasinophytes**	0.066±0.032	0.095±0.062	0.040±0.027	1.7	2.4
**Depth @ 5 m [mg m**^**-3**^**]**	**TChl *a***	0.211±0.050^***^	0.121±0.030	0.082±0.021	2.6	1.5
	**Dinoflagellates**	0.006±0.003^***^	0.002±0.001	0.002±0.001	3	1
	**Diatoms**	<0.001	<0.001	<0.001	\	\
	**Haptophytes_8**	0.022±0.010^*^	0.014±0.010	0.008±0.003	2.8	1.8
	**Haptophytes_6**	0.013±0.006^**^	0.007±0.004	0.003±0.001	4.3	2.3
	***Prochlorococcus***	0.014±0.007^**^	0.010±0.004	0.005±0.004	2.8	2
	***Synechococcus***	0.144±0.035^***^	0.082±0.022	0.064±0.015	2.3	1.3
	**Prasinophytes**	0.003±0.003	0.003±0.008	<0.001	\	\

Three comparing means, including the water column integration, the DCML and the surface layer (at 5 m) were introduced. The OUT group was treated as the control in the multiple comparison using one-way ANOVA.

* implied significant difference in the level of p<0.050

** for p<0.010

*** for p<0.001.

The IN/OUT and EDGE/OUT implied the multiple or fraction of IN and EDGE compared to OUT.

### Vertical profiles of the phytoplankton community

The results of CHEMTAX implied that diatoms, haptophytes_8, prasinophytes, *Synechococcus* and *Prochlorococcus* contributed over 85% of the TChl *a* biomass in the survey region. The vertical distribution of TChl *a* biomass for the major phytoplankton groups were exhibited in Transect Y1 (Fig 5). Most of the phytoplankton groups had the highest biomass at the DCML. Most of the phytoplankton groups had similar vertical distribution patterns except *Synechococcus* (Fig 5D). The TChl *a* biomass of *Synechococcus* was relatively higher in the upper mixed layer throughout the water column. Haptophytes_8 (Fig 5E) and prasinophytes (Fig 5C) distributed throughout the DCML and the former dominated over the other phytoplankton groups at the DCML. The highest concentration patch of diatoms (Fig 5B) existed only at the center of the C2, in contrast, *Prochlorococcus* (Fig 5F) was observed throughout the DCML except at the center of C2.

By comparing the biomass of each phytoplankton group throughout the water column of the C2 and the ambient water (Table 2), it could be found that the water column integrated biomass of diatoms was 21.3-fold higher at IN (p<0.001) relative to its biomass at OUT. The integrated *Synechococcus* biomass at IN also had ~60% amplification than that at OUT (p<0.050). In contrast, the biomass of *Prochlorococcus* was the most abundant at OUT, but was reduced ~80% at IN (p<0.001) and ~50% at EDGE (p<0.010).

At the DCML, there was no significant difference in the total phytoplankton biomass among the three blocks, but the community compositions showed remarkable differences (Table 2). For the IN group, the 25.5-fold increase in biomass for diatoms (p<0.050), 7.1-fold for *Synechococcus* (p<0.001), and 1.8-fold for dinoflagellates (p<0.050) was remarkable. This was similar to the water column integration, where the biomass of *Prochlorococcus* at IN (p<0.010) was only 20% of the richness at OUT, and showed a 50% shrink at EDGE (p<0.050). Even more, the dominant group, haptophytes_8 decreased half of its biomass at IN compared to OUT (p<0.010). Therefore, the dominant groups changed from haptophytes_8 and *Prochlorococcus* at OUT and EDGE to haptophytes_8 and *Synechococcus* within the C2.

The greatest variation of phytoplankton composition was at the surface layer. Almost all phytoplankton groups responded positively within the C2 to build a 2.6-fold TChl *a* enhancement (p<0.001), in which the biomass of dinoflagellates increased 3.0-fold (p<0.001), haptophytes_8 increased 2.8-fold (p<0.050), haptophytes_6 increased 4.3-fold (p<0.010) and *Synechococcus* increased 2.3-fold (p<0.001) at IN compared to OUT. The biomass of *Prochlorococcus*, also increased 2.8-fold (p<0.010) at IN than the OUT.

**Fig 7:**
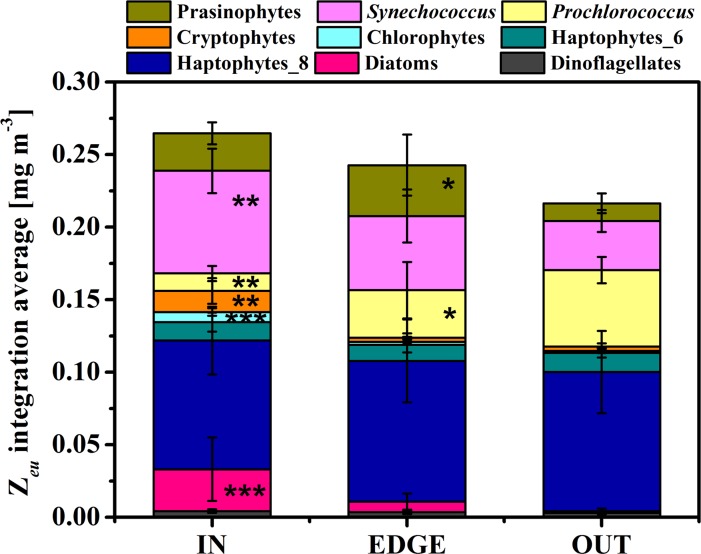
The water column integrated phytoplankton group composition in term of group-average TChl *a* biomass within the euphotic zone [mg m^-3^]. The group OUT was treated as the control in the multiple comparison using one-way ANOVA. * implied significantly different in the level of p<0.050, ** for p<0.010 and *** for p<0.001.

Because the patterns of TChl *a* biomass at the surface layer were not consistent with those integrated over the water column, TChl *a* integrated over the euphotic layer was estimated and the depth-average value of TChl *a* inventory was calculated to eliminate the difference of euphotic depth. For IN and EDGE, the Z_*eu*_ integrated TChl *a* biomass was ~95% and ~86% of biomass integrated over the upper 100 m. For the Z_*eu*_ integration average, 22% for IN and 12% for EDGE TChl *a* biomass enhancement was obvious but had no significant difference (p>0.050). The growth or decline for each community group was similar as the upper 100 m water column integration (Fig 7).

## Discussion

### Physical-biological coupling in a cyclonic eddy

The upper layer oceanic circulation in the western SCS off the Vietnam coast is mainly driven by monsoons. Under the effects of the southwest monsoon and complex topography, this area is characterized by high activities of mesoscale eddies in summer [21]. During the period of the summer monsoon, a pair of mesoscale eddies was observed in our study area, the C2 in the north and the AC2 in the southeast [27,28]. In the cyclonic eddy, it was expected that the uplifting of pycnocline would bring nutrients from the twilight zone to the euphotic zone. And then the nutrient pumping induced the increasing in phytoplankton biomass. The isopycnal sigma-*t* = 24 kg m^-3^ almost overlapped with the baseline of euphotic zone in C2, which was uplifted to about 50~80 m, in comparison with the 93.2±18.6 m in the surrounding water. On the other hand, the DCML adhered to isopycnal sigma-*t* = 22 kg m^-3^, which was deeper than 75 m outside the eddy, and at about 25 m in the center of C2. The doming isopycnal therefore brought deep water with higher nutrients concentration and the light-limited phytoplankton individuals to the euphotic zone, resulting in rapid growth and biomass accumulation of phytoplankton. The observed phytoplankton TChl *a* biomass throughout the water column (Fig 7, the Z_*eu*_ integrated TChl *a*: IN > EDGE > OUT) in the C2 had a notable trend of increasing phytoplankton biomass in the euphotic zone. It could be explained by the mechanism that phytoplankton growth was stimulated by upwelling nutrients in the cyclonic eddy. However in the present study, this mechanism was impractical from the perspective of water column integration and the DCML (Table 2). One of the explanation might be the effects of eddy advection and diffusion. Under the effect of the rotation and gradient of potential density between the cyclonic eddy center and adjacent water, horizontal advection of TChl *a* concentration emerged in the edge of eddy [6,42]. In many of the previous studies, the eddy edge was account as a part of the eddy, which would cause the leveling effect on biomass. Without consideration of horizontal advection effects, the spatial difference of the biomass between the center and edge was usually neglected [6]. At the same time, the diffusion from nutricline provided nutrients for phytoplankton growth in the upper mixed layer. Thereby the mixing stimulated the phytoplankton biomass at the surface layer in the center of C2. So it was a particularity that the biological signal was more obvious than the hydrologic characters in the surface layer in this study.

Even though there were no significantly differences of TChl *a* biomass among the three blocks of C2, neither at water column integration nor at the DCML, the variations in phytoplankton community composition were very evident (Table 2). Haptophytes_8, mainly *Phaeocystis* spp. and *Dicrateria* spp., containing 19’-but-fucoxanthin and fractional 19’-hex-fucoxanthin simultaneously [39,43], were the dominant group at the DCML both inside and outside C2, but decreased by about half inside the C2 (p<0.050). This scenarios were similar to what in the cyclonic eddies “*Mikalele*” and “*Loretta*” [44], but also some different circumstances for other phytoplankton population. In the present study, diatoms and *Synechococcus* at the DCML within the eddy showed a positive response to nutrient enrichment, with a 25.5-fold (p<0.050) and a 7.1-fold (p<0.001) increase of TChl *a* biomass, respectively. *Prochlorococcus* decreased sharply in the center of C2 to only 20% of the status outside C2 (p<0.010). In the upper mixed layer, most of phytoplankton groups, except the diatoms, exhibited positive response to nutrient enrichment in the center of C2 (Table 2). The different scenario between “*Mikalele*”, “*Loretta*” and C2 in this study might be attributed to the different physical conditions. In view of “*Mikalele*” and “*Loretta*”, even the eddies in the same area which were induced simultaneity had different expression on the Chl *a* enhancement. For the elder one “*Loretta*”, the Chl *a* concentration at DCML was 2-fold higher than what in the younger one “*Mikalele*” [44]. Although the thermal gradients at surface in “*Mikalele*” were stronger than the elder one, it was converse at sub-surface layer. Accordingly, the dome structure of nutricline showed more concentrated and sharper in the elder “*Loretta*”. Similarly, the C2 in this study had the loose thermal gradient and nutricline at the sub-surface layer, and the response of phytoplankton community was similar to “*Mikalele*”.

“*Opal*” was a cyclonic eddy in the lee of the Hawaiian Islands [5,45]. It also had sharply uplifted isopycnals in the eddy center where could be 80~100 m shallower than the surrounding water [5]. Because of the intense nutrients pumping, the responses of phytoplankton community in “*Opal*” showed little similarity to C2. For instance, diatoms at the DCML (70~90 m) in “*Opal*” showed a 100-fold increase and formed a diatom bloom. It was homologous in phytoplankton composition in the upper mixed layer, where was dominated by pico-phytoplankton both inside and outside eddy [5,45]. On the other hand, none of the other cyclonic eddies in the lee of Hawaii showed an increase in diatoms, but instead a modest increase of non-diatom eukaryotes (nano-plankton) [9–11,44,46–48]. The diatoms species succession inside “*Opal*” was dramatic during the bloom, from larger chain species to smaller cells [5]. In general, the development of a diatom-dominated community in a large nutrient pulse eddy, on the contrary, a pico- and nano-phytoplankton dominated community in an eddy with continuous low nutrient supply [5]. But it also depended on the constituent of upwelled nutrient, especially the silicate/nitrate ratios (Si*) [49]. According to Bibby and Moore (2011), the pico-phytoplankton or diatoms dominance was directed by leanness or richness of silicate in the cyclonic eddy or mode water eddy in the Sargasso Sea. And the higher Si* in the eddy cases in the Hawaii originated from the North Pacific Intermediate Water (NPIW) and supplied the sustaining diatoms biomass in the cyclonic eddy [49]. In the present study, the open-bottom and closed–core nutrient was continuously supplied from the uplifted isopycnals inside C2, where there was absent of a significant effect on phytoplankton biomass and composition at the DCML. It was a regret that the lack of silicate concentration data in this study. But there were diatom patchiness at the DCML in the C2 center, it might be a signal of nutrient input from the deep zone. In contrast, the pico- and nano-phytoplankton community in the upper mixed zone demonstrated higher biomass which due to stimulation by nutrients brought up from the deep zone.

Among the prokaryote pico-phytoplankton, *Synechococcus* dominated in the upper mixed zone of the water column, especially within the C2. *Prochlorococcus* became relatively important at the DCML outside the C2, and nearly disappeared in the center of C2. The shift from dominance of *Synechococcus* to negligible *Prochlorococcus* biomass at the DCML inside C2 was probably a reflection of the combined effects of nutrient pumping and microzooplankton grazing processes [29].

### Nutrient pumping induced phytoplankton biomass promotion at the C2

**Fig 8:**
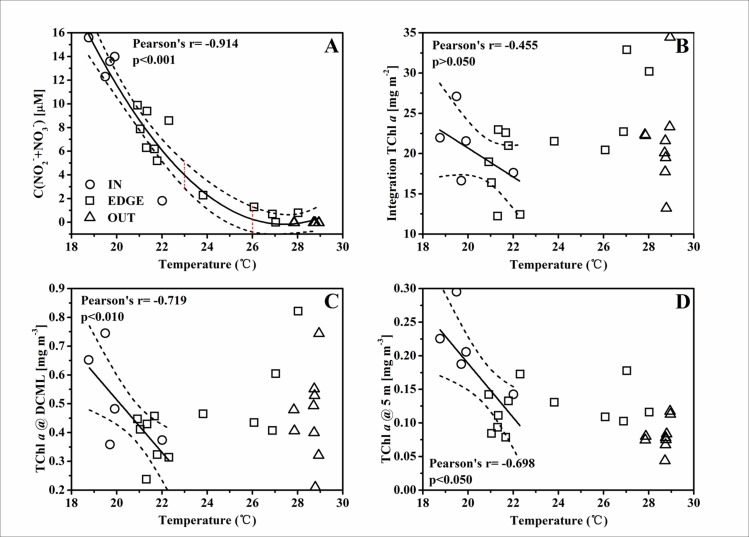
**The linear relationship between (A) NO**_**2**_^**-**^**+NO**_**3**_^**-**^ **[**μ**M], (B) TChl *a* biomass of the upper 100 m water column integration [mg m**^**-2**^**], (C) TChl *a* biomass at DCML [mg m**^**-3**^**], (D) TChl *a* biomass at surface layer [mg m**^**-3**^**] and the temperature at the 50 m depth layer.** The two red dashed lines in Fig 8A indicate 23.0°C and 26.0°C, which were the frontal temperature gradients. The solid line shows the linear regression, and the two dashed lines the 95% upper confidence limit and lower confidence limit.

**Fig 9:**
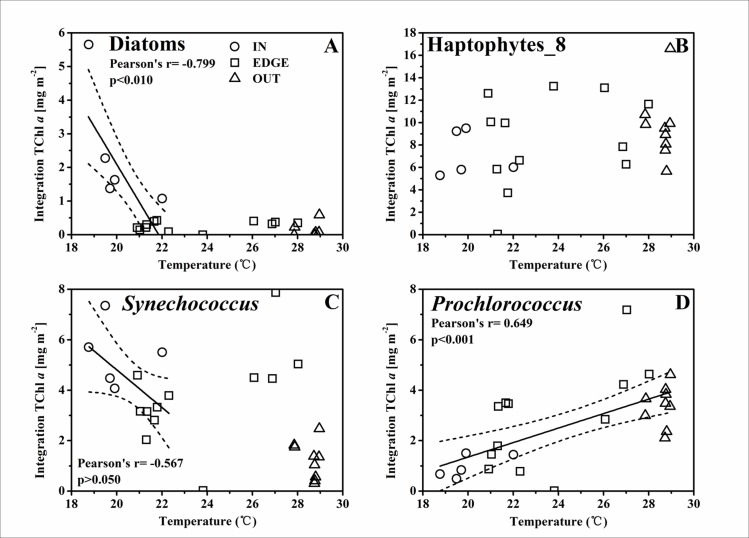
**The linear relationship between the TChl *a* biomass in the upper 100 m water column integration of (A) diatoms [mg m**^**-2**^**], (B) haptophytes_8 [mg m**^**-2**^**], (C) *Synechococcus* [mg m**^**-2**^**], (D) *Prochlorococcus* [mg m**^**-2**^**] and the temperature at the 50 m depth layer.** The solid line and two dashed lines are the same as in Fig 8.

**Fig 10:**
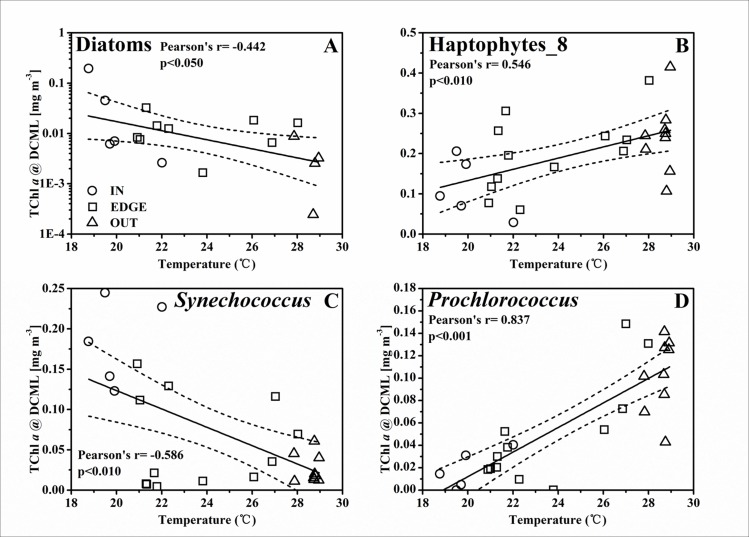
**The linear relationship between the TChl *a* biomass at the DCML of (A) diatoms [mg m**^**-3**^**], (B) haptophytes_8 [mg m**^**-3**^**], (C) *Synechococcus* [mg m**^**-3**^**], (D) *Prochlorococcus* [mg m**^**-3**^**] and the temperature at the 50 m depth layer.** The solid line and two dashed lines are the same as in Fig 8.

**Fig 11:**
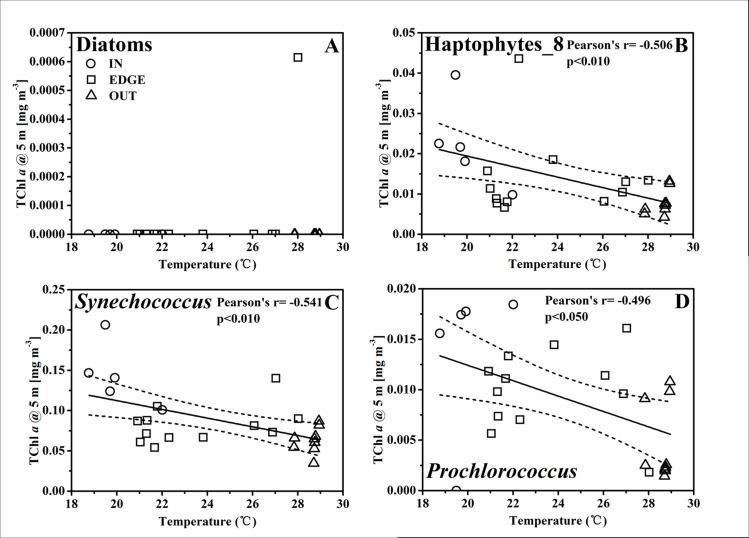
**The linear relationship between the TChl *a* biomass at the surface layer of (A) diatoms [mg m**^**-3**^**], (B) haptophytes_8 [mg m**^**-3**^**], (C) *Synechococcus* [mg m**^**-3**^**], (D) *Prochlorococcus* [mg m**^**-3**^**] and the temperature at the 50 m depth layer.** The solid line and two dashed lines are the same as in Fig 8.

The highly heterogeneous character of the physical properties in cyclonic eddy C2 [28] probably influenced the distribution of nutrients, therefore resulting in a significant spatial variation of phytoplankton biomass within the eddy. In this study, the temperature at the 50 m layer was used as a proxy of the nutrients pumping strength in C2. The concentration of NO_2_^-^+NO_3_^-^ (unpublished data from Dai) was remarkably higher in IN than in EDGE and OUT (Fig 4D & 4E). The remarkable negative correlation between the concentration of NO_2_^-^+NO_3_^-^ and the temperature at 50 m implied the gliding of nutrient gradients from the affluence eddy center to the barren adjacent water (Fig 8A). Furthermore, the thermal gradient between 23.0~26.0°C with few dot implied that the C2 front was relatively narrow, in spite of no sharper than the cyclone “*Loretta*” [44] and “*Opal*” [5]. The nutrients pumping from the deep water could induce a response of phytoplankton biomass, and hence there was a negative relationship between spatial variability in the total phytoplankton biomass and the temperature at 50 m (Fig 8). Thus, the temperature at 50 m was used as a proxy to represent the pumping intensity, as well as its intrinsic effect on the response of different phytoplankton groups (Figs 9–11). There were negative correlations between diatoms, *Synechococcus* and the temperature for the TChl *a* inventory (Fig 9). Negative relationships of diatoms and *Synechococcus* spp. with temperature at the DCML were also presented, while the haptophytes_8 and *Prochlorococcus* behaved positively with temperature (Fig 10). In the upper mixed zone (Fig 11), the response of phytoplankton to the upwelled nutrients was very notable as compared with the status at the DCML, including haptophytes_8 and *Synechococcus* spp. Diatoms showed a good positive response at the DCML and no response in the upper mixed zone.

From these results, we concluded that the C2 was a mesoscale cyclonic eddy in which pico- and nano-phytoplankton were the predominant groups within and outside. A weak but continuous nutrient supply from the uplifting isopycnals within the eddy stimulated the pico- and nano-phytoplankton (low V_max_, half-saturation constant for nutrient uptake) in the upper mixed zone, whereas the increase of phytoplankton biomass was not significant at the DCML, although the diatoms TChl *a* biomass responded positively to the upwelled nutrients (high V_max_) [33,50].

The present study analyzed the phytoplankton community influenced by a cyclonic eddy in the western SCS using pigments-CHEMTAX methods. It could be seen that the eddy C2 affected the phytoplankton TChl *a* biomass through elevating the water column inventory biomass. The doming of the DCML made it thinner, denser and shallower, but the phytoplankton biomass did not respond so remarkable, even though the community composition had different responses to the C2. Three distribution patterns were classified according to their vertical manner and biomass. *Synechococcus* at the surface, haptophytes_8, diatoms and prasinophytes at the DCML played key roles in enhancing the water column inventory biomass. The *Prochlorococcus* decreased dramatically at DCML in C2 center. Although the hydrological and nutrient signals of the C2 were not so obvious at the surface, the TChl *a* and almost all the phytoplankton populations showed remarkable responses to C2. The water mixing of the upper 20 m upon the nutricline might be responsible for sustaining the nutrient source, while the deficit of nutrients meant that it was still oligotrophic as a result of their depletion by the phytoplankton.

## Supporting Information

S1 Datasets The pigments concentration for each sampling layer at every station. (XLSX)

S2 Datasets The group-specific phytoplankton contribution to the TChl *a* biomass calculated by CHEMTAX. (XLSX)

S1 Fig The profiles of fluorescence (in black) and TChl *a* concentration (in blue) at sta. Y10 (left), sta. Y13 (middle) and sta. Y14 (right). (TIF)

S2 Fig Geostrophic current measured by an ADCP at 57 m (left) and at 73 m (right) in the western SCS in September 2007. (TIF)

S3 Fig The time series variation of SLA for IN (in blue), EDGE (in yellow) and OUT (in red) in the study area. (TIF)

S1 Text Note for the CHEMTAX running in this study. (DOCX)
