# Supplementary figures and images for: Physical-Biological Coupling in the Western South China Sea: The Response of Phytoplankton Community to a Mesoscale Cyclonic Eddy
Source: PLoS One. 2016 Apr 18;11(4):e0153735. doi: 10.1371/journal.pone.0153735 (PMC4835056; doi:10.1371/journal.pone.0153735)

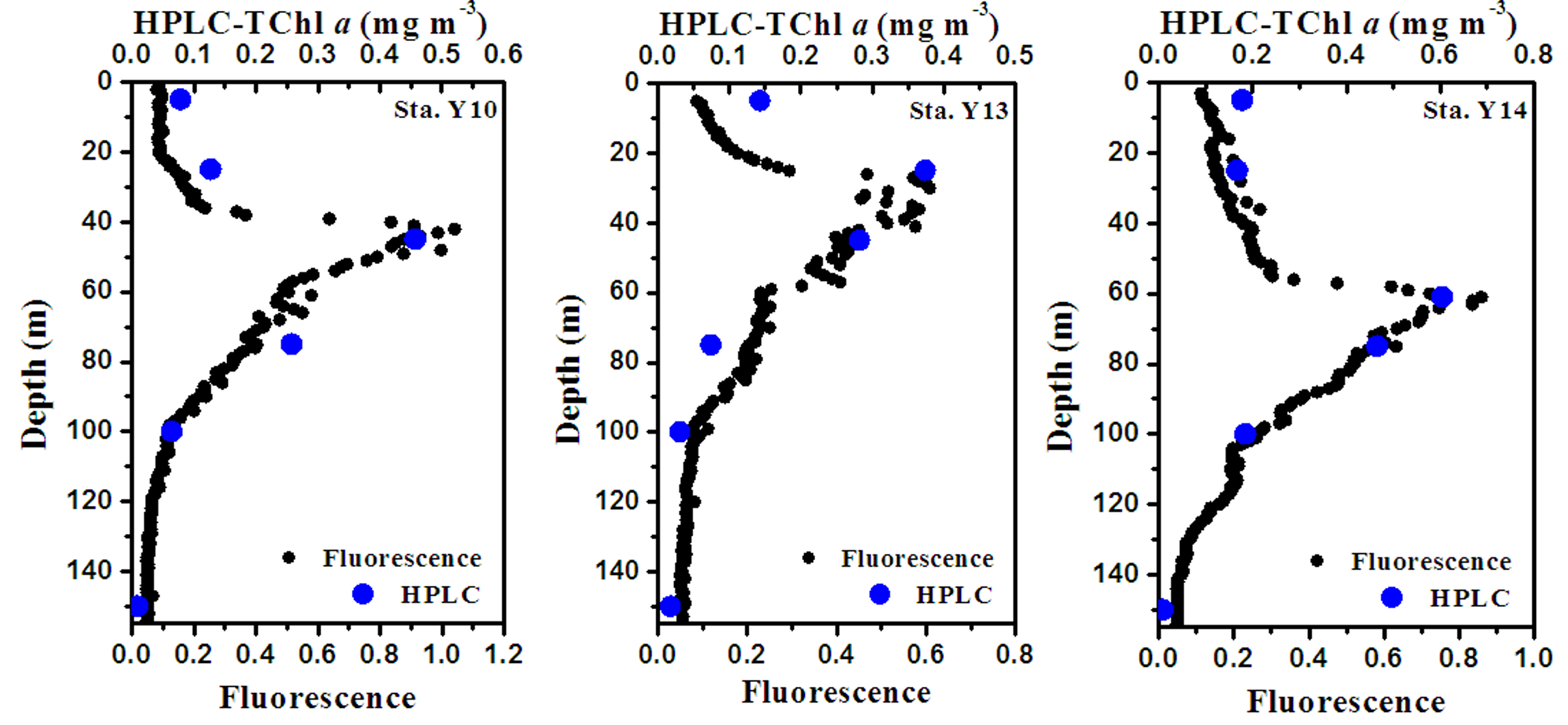

Supplement: S1 Fig — (TIF) [file pone.0153735.s003.tif]

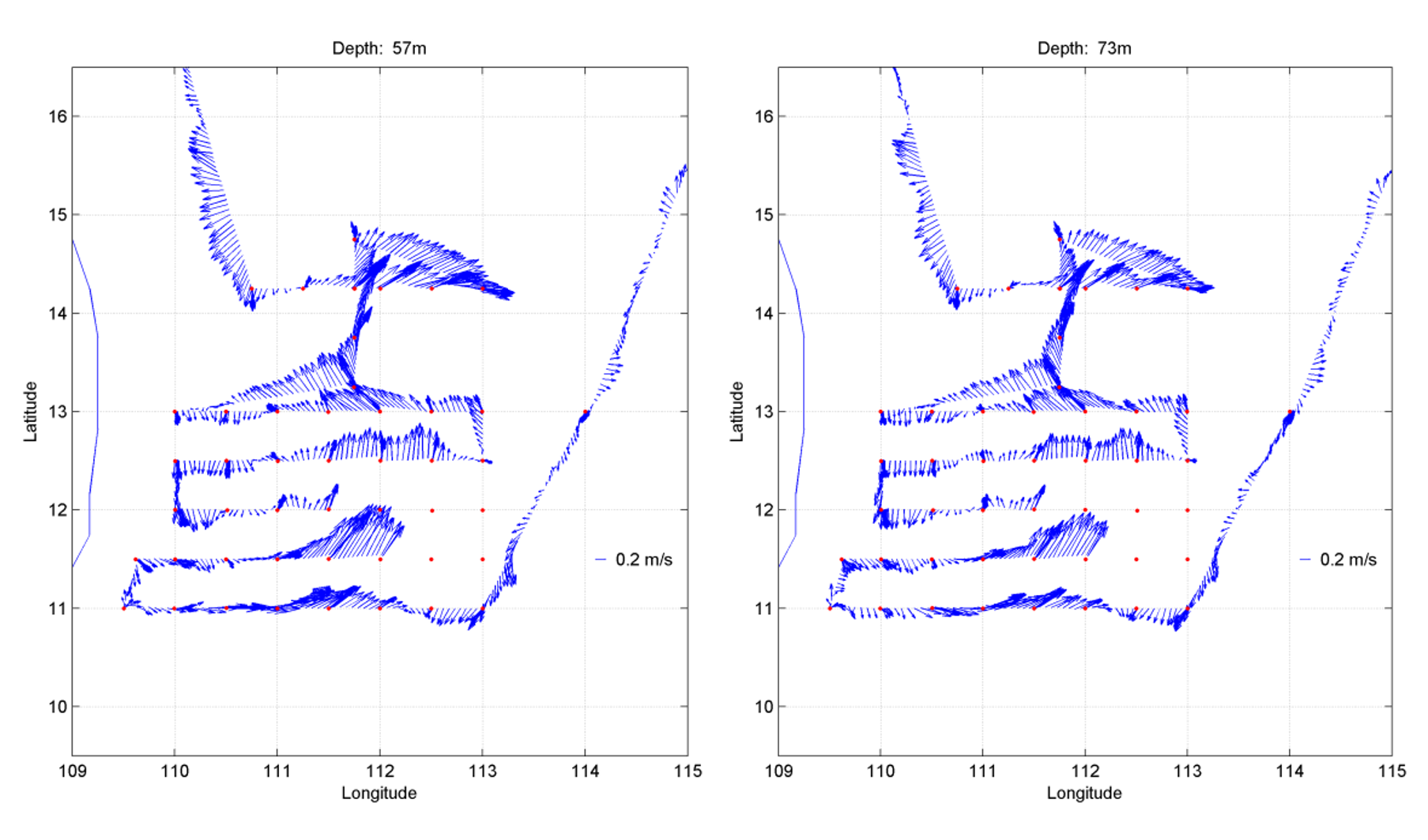

Supplement: S2 Fig — (TIF) [file pone.0153735.s004.tif]

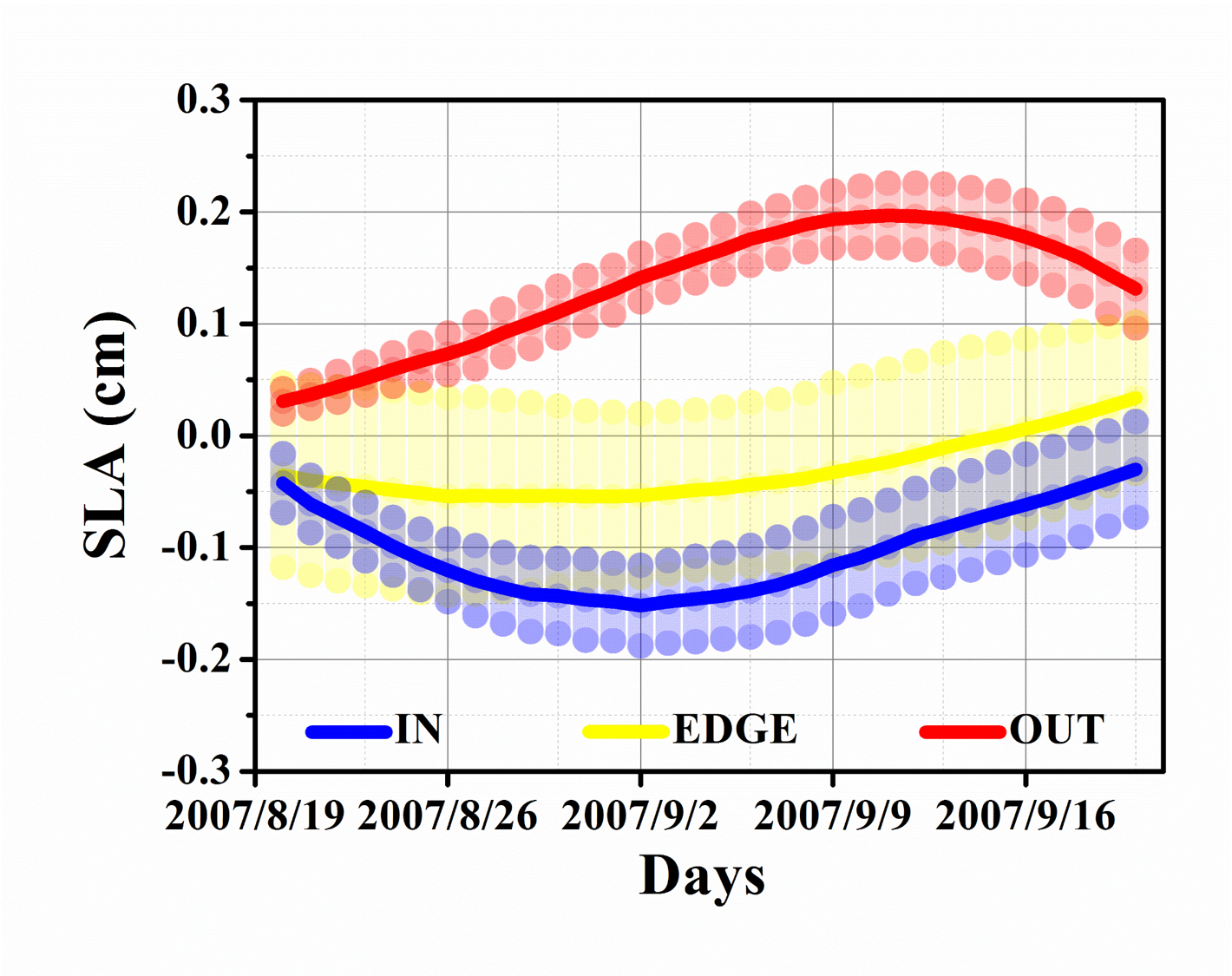

Supplement: S3 Fig — (TIF) [file pone.0153735.s005.tif]
