# Supplementary material for: Physical-Biological Coupling in the Western South China Sea: The Response of Phytoplankton Community to a Mesoscale Cyclonic Eddy
Source: PLoS One. 2016 Apr 18;11(4):e0153735. doi: 10.1371/journal.pone.0153735 (PMC4835056; doi:10.1371/journal.pone.0153735)
Supplement: S1 Text — (DOCX) [file pone.0153735.s006.docx]

**Note for the CHEMTAX running**

The procedure of the CHEMTAX running for this study which included the initial inputting pigment/Chl *a* ratio for the SCS (modified through the ratio matrix for Southern Ocean in Mackey *et al*. 1996 with additional *Prochlorococcus* and Dv-Chl *a*), and the variation of the outputting pigment/Chl *a* ratio after the successive running of CHEMTAX according to Latasa 2007. The essential of the data grouping based on the light-depth which were divided into 6 groups, including 0~10 m, 11~30 m, 31~60 m, 61~90 m, 91~130 m and 131~150 m group. The final results were the fifth running of the CHEMTAX since the convergent ratios were better than running once and the other running times (evaluated by the regression between the calculation values and the measured values).

**Table-The initial inputting pigment/Chl *a* ratio in this study.**

|  | Peri | But-Fuco | Fuco | Hex-Fuco | Neo | Pras | Viol | Allo | Lut | Zea | Chl *b* | Dv-Chl *a* | Chl *a* |
| --- | --- | --- | --- | --- | --- | --- | --- | --- | --- | --- | --- | --- | --- |
| Dino | 1.06 |  |  |  |  |  |  |  |  |  |  |  | 1.00 |
| Diat |  |  | 0.75 |  |  |  |  |  |  |  |  |  | 1.00 |
| Hapt (T8) |  | 0.25 | 0.59 | 0.54 |  |  |  |  |  |  |  |  | 1.00 |
| Hapt (T6) |  |  |  | 1.70 |  |  |  |  |  |  |  |  | 1.00 |
| Chlo |  |  |  |  | 0.06 |  | 0.06 |  | 0.20 | 0.01 | 0.26 |  | 1.00 |
| Cryp |  |  |  |  |  |  |  | 0.23 |  |  |  |  | 1.00 |
| Proc |  |  |  |  |  |  |  |  |  | 0.37 | 0.68 | 1.00 |  |
| Syne |  |  |  |  |  |  |  |  |  | 0.35 |  |  | 1.00 |
| Pras |  |  |  |  | 0.15 | 0.32 | 0.06 |  | 0.01 |  | 0.95 |  | 1.00 |

**Table-The fifth running output pigment/Chl *a* ratio for 0~10 group.**

|  | Peri | But-Fuco | Fuco | Hex-Fuco | Neo | Pras | Viol | Allo | Lut | Zea | Chl *b* | Dv-Chl *a* | Chl *a* |
| --- | --- | --- | --- | --- | --- | --- | --- | --- | --- | --- | --- | --- | --- |
| Dino | 1.06 | 0 | 0 | 0 | 0 | 0 | 0 | 0 | 0 | 0 | 0 | 0 | 1 |
| Diat | 0 | 0 | 0.75 | 0 | 0 | 0 | 0 | 0 | 0 | 0 | 0 | 0 | 1 |
| Hapt (T8) | 0 | 0.25 | 0.59 | 0.54 | 0 | 0 | 0 | 0 | 0 | 0 | 0 | 0 | 1 |
| Hapt (T6) | 0 | 0 | 0 | 1.7 | 0 | 0 | 0 | 0 | 0 | 0 | 0 | 0 | 1 |
| Chlo | 0 | 0 | 0 | 0 | 0.06 | 0 | 0.06 | 0 | 0.2 | 0.01 | 0.26 | 0 | 1 |
| Cryp | 0 | 0 | 0 | 0 | 0 | 0 | 0 | 0.23 | 0 | 0 | 0 | 0 | 1 |
| Proc | 0 | 0 | 0 | 0 | 0 | 0 | 0 | 0 | 0 | 0.37 | 0.68 | 1 | 0 |
| Syne | 0 | 0 | 0 | 0 | 0 | 0 | 0 | 0 | 0 | 0.35 | 0 | 0 | 1 |
| Pras | 0 | 0 | 0 | 0 | 0.15 | 0.32 | 0.06 | 0 | 0.01 | 0 | 0.95 | 0 | 1 |

**Table-The fifth running output pigment/Chl *a* ratio for 11~30 group.**

|  | Peri | But-Fuco | Fuco | Hex-Fuco | Neo | Pras | Viol | Allo | Lut | Zea | Chl *b* | Dv-Chl *a* | Chl *a* |
| --- | --- | --- | --- | --- | --- | --- | --- | --- | --- | --- | --- | --- | --- |
| Dino | 1.06 | 0 | 0 | 0 | 0 | 0 | 0 | 0 | 0 | 0 | 0 | 0 | 1 |
| Diat | 0 | 0 | 0.75 | 0 | 0 | 0 | 0 | 0 | 0 | 0 | 0 | 0 | 1 |
| Hapt (T8) | 0 | 0.06 | 0.06 | 0.1 | 0 | 0 | 0 | 0 | 0 | 0 | 0 | 0 | 1 |
| Hapt (T6) | 0 | 0 | 0 | 1.7 | 0 | 0 | 0 | 0 | 0 | 0 | 0 | 0 | 1 |
| Chlo | 0 | 0 | 0 | 0 | 0.06 | 0 | 0.06 | 0 | 0.2 | 0.01 | 0.26 | 0 | 1 |
| Cryp | 0 | 0 | 0 | 0 | 0 | 0 | 0 | 0.23 | 0 | 0 | 0 | 0 | 1 |
| Proc | 0 | 0 | 0 | 0 | 0 | 0 | 0 | 0 | 0 | 0.29 | 0.15 | 1 | 0 |
| Syne | 0 | 0 | 0 | 0 | 0 | 0 | 0 | 0 | 0 | 2.22 | 0 | 0 | 1 |
| Pras | 0 | 0 | 0 | 0 | 0.14 | 0.31 | 0.06 | 0 | 0.01 | 0 | 1.39 | 0 | 1 |

**Table-The fifth running output pigment/Chl *a* ratio for 31~60 group.**

|  | Peri | But-Fuco | Fuco | Hex-Fuco | Neo | Pras | Viol | Allo | Lut | Zea | Chl *b* | Dv-Chl *a* | Chl *a* |
| --- | --- | --- | --- | --- | --- | --- | --- | --- | --- | --- | --- | --- | --- |
| Dino | 1.16 | 0 | 0 | 0 | 0 | 0 | 0 | 0 | 0 | 0 | 0 | 0 | 1 |
| Diat | 0 | 0 | 1.75 | 0 | 0 | 0 | 0 | 0 | 0 | 0 | 0 | 0 | 1 |
| Hapt (T8) | 0 | 0.1 | 0.04 | 0.14 | 0 | 0 | 0 | 0 | 0 | 0 | 0 | 0 | 1 |
| Hapt (T6) | 0 | 0 | 0 | 5.46 | 0 | 0 | 0 | 0 | 0 | 0 | 0 | 0 | 1 |
| Chlo | 0 | 0 | 0 | 0 | 0.06 | 0 | 0.06 | 0 | 0.05 | 0.01 | 0.26 | 0 | 1 |
| Cryp | 0 | 0 | 0 | 0 | 0 | 0 | 0 | 0.23 | 0 | 0 | 0 | 0 | 1 |
| Proc | 0 | 0 | 0 | 0 | 0 | 0 | 0 | 0 | 0 | 0.23 | 0.3 | 1 | 0 |
| Syne | 0 | 0 | 0 | 0 | 0 | 0 | 0 | 0 | 0 | 4.33 | 0 | 0 | 1 |
| Pras | 0 | 0 | 0 | 0 | 0.14 | 0.32 | 0.09 | 0 | 0.01 | 0 | 1.81 | 0 | 1 |

**Table-The fifth running output pigment/Chl *a* ratio for 61~90 group.**

|  | Peri | But-Fuco | Fuco | Hex-Fuco | Neo | Pras | Viol | Allo | Lut | Zea | Chl *b* | Dv-Chl *a* | Chl *a* |
| --- | --- | --- | --- | --- | --- | --- | --- | --- | --- | --- | --- | --- | --- |
| Dino | 1.06 | 0 | 0 | 0 | 0 | 0 | 0 | 0 | 0 | 0 | 0 | 0 | 1 |
| Diat | 0 | 0 | 0.75 | 0 | 0 | 0 | 0 | 0 | 0 | 0 | 0 | 0 | 1 |
| Hapt (T8) | 0 | 0.17 | 0.05 | 0.14 | 0 | 0 | 0 | 0 | 0 | 0 | 0 | 0 | 1 |
| Hapt (T6) | 0 | 0 | 0 | 1.7 | 0 | 0 | 0 | 0 | 0 | 0 | 0 | 0 | 1 |
| Chlo | 0 | 0 | 0 | 0 | 0.06 | 0 | 0.19 | 0 | 0.06 | 0.01 | 0.26 | 0 | 1 |
| Cryp | 0 | 0 | 0 | 0 | 0 | 0 | 0 | 0.23 | 0 | 0 | 0 | 0 | 1 |
| Proc | 0 | 0 | 0 | 0 | 0 | 0 | 0 | 0 | 0 | 0.1 | 0.08 | 1 | 0 |
| Syne | 0 | 0 | 0 | 0 | 0 | 0 | 0 | 0 | 0 | 0.4 | 0 | 0 | 1 |
| Pras | 0 | 0 | 0 | 0 | 0.08 | 0.13 | 0.05 | 0 | 0.01 | 0 | 2.14 | 0 | 1 |

**Table-The fifth running output pigment/Chl *a* ratio for 91~130 group.**

|  | Peri | But-Fuco | Fuco | Hex-Fuco | Neo | Pras | Viol | Allo | Lut | Zea | Chl *b* | Dv-Chl *a* | Chl *a* |
| --- | --- | --- | --- | --- | --- | --- | --- | --- | --- | --- | --- | --- | --- |
| Dino | 1.06 | 0 | 0 | 0 | 0 | 0 | 0 | 0 | 0 | 0 | 0 | 0 | 1 |
| Diat | 0 | 0 | 0.75 | 0 | 0 | 0 | 0 | 0 | 0 | 0 | 0 | 0 | 1 |
| Hapt (T8) | 0 | 0.18 | 0.05 | 0.08 | 0 | 0 | 0 | 0 | 0 | 0 | 0 | 0 | 1 |
| Hapt (T6) | 0 | 0 | 0 | 1.7 | 0 | 0 | 0 | 0 | 0 | 0 | 0 | 0 | 1 |
| Chlo | 0 | 0 | 0 | 0 | 0.06 | 0 | 0.18 | 0 | 0.03 | 0.01 | 0.26 | 0 | 1 |
| Cryp | 0 | 0 | 0 | 0 | 0 | 0 | 0 | 0.23 | 0 | 0 | 0 | 0 | 1 |
| Proc | 0 | 0 | 0 | 0 | 0 | 0 | 0 | 0 | 0 | 0.13 | 0.07 | 1 | 0 |
| Syne | 0 | 0 | 0 | 0 | 0 | 0 | 0 | 0 | 0 | 2.71 | 0 | 0 | 1 |
| Pras | 0 | 0 | 0 | 0 | 0.06 | 0.06 | 0.05 | 0 | 0.01 | 0 | 2.26 | 0 | 1 |

**Table-The fifth running output pigment/Chl *a* ratio for 131~150 group.**

|  | Peri | But-Fuco | Fuco | Hex-Fuco | Neo | Pras | Viol | Allo | Lut | Zea | Chl *b* | Dv-Chl *a* | Chl *a* |
| --- | --- | --- | --- | --- | --- | --- | --- | --- | --- | --- | --- | --- | --- |
| Dino | 1.06 | 0 | 0 | 0 | 0 | 0 | 0 | 0 | 0 | 0 | 0 | 0 | 1 |
| Diat | 0 | 0 | 0.81 | 0 | 0 | 0 | 0 | 0 | 0 | 0 | 0 | 0 | 1 |
| Hapt (T8) | 0 | 0.22 | 0.06 | 0.05 | 0 | 0 | 0 | 0 | 0 | 0 | 0 | 0 | 1 |
| Hapt (T6) | 0 | 0 | 0 | 1.7 | 0 | 0 | 0 | 0 | 0 | 0 | 0 | 0 | 1 |
| Chlo | 0 | 0 | 0 | 0 | 0.06 | 0 | 0.07 | 0 | 0.03 | 0.01 | 0.26 | 0 | 1 |
| Cryp | 0 | 0 | 0 | 0 | 0 | 0 | 0 | 0.23 | 0 | 0 | 0 | 0 | 1 |
| Proc | 0 | 0 | 0 | 0 | 0 | 0 | 0 | 0 | 0 | 0.16 | 0.19 | 1 | 0 |
| Syne | 0 | 0 | 0 | 0 | 0 | 0 | 0 | 0 | 0 | 0.35 | 0 | 0 | 1 |
| Pras | 0 | 0 | 0 | 0 | 0.12 | 0.1 | 0.06 | 0 | 0.01 | 0 | 1.68 | 0 | 1 |

**Figure-Pigment/TChl *a* ratios after successive running of CHEMTAX in the SCS.** a. Allo/TChl *a* for Cryp, b. Zea/TChl *a* for Chlo, c. Hex-Fuco/TChl *a* for Hapt_8, d. Pras/TChl *a* for Pras, e. Zea/TChl *a* for Proc, and f. Fuco/TChl *a* for Diat*.*

**Figure-Comparison of the pigment concentration (ng/L) between the measured value (True) and the results of the fifth running (estimated).** a. But-Fuco and b. Chl *b.*
